# Predicting mechanical properties of material extrusion additive manufacturing-fabricated structures with limited information

**DOI:** 10.1038/s41598-022-19053-3

**Published:** 2022-08-30

**Authors:** Amy M. Peterson, David O. Kazmer

**Affiliations:** Department of Plastics Engineering, University of Massachusetts Lowell, One University Ave, Lowell, MA 01854 USA

**Keywords:** Mechanical engineering, Polymers, Computational methods

## Abstract

Mechanical properties of additively manufactured structures fabricated using material extrusion additive manufacturing are predicted through combining thermal modeling with entanglement theory and molecular dynamics approaches. A one-dimensional model of heat transfer in a single road width wall is created and validated against both thermography and mechanical testing results. Various model modifications are investigated to determine which heat transfer considerations are important to predicting properties. This approach was able to predict tear energies on reasonable scales with minimal information about the polymer. Such an approach is likely to be applicable to a wide range of amorphous and low crystallinity thermoplastics.

## Introduction

Material extrusion (MatEx) additive manufacturing (AM) consists of selective dispensing of a material through a nozzle or orifice. Fused filament fabrication (FFF), which is a desktop form of MatEx in which a feedstock in the form of a polymer-based filament is heated until flowable and extruded through a rastering nozzle onto a print surface, is the most common form of MatEx as well as the most widely used form of AM generally. Polymer filaments represent 19.7% of the 2020 AM materials market with estimated sales of $414.1 million^1^. Structures fabricated using FFF are commonly used industrially for functional prototyping and tooling, with broader adoption often limited by inferior mechanical properties. In FFF in particular^2^, molten polymer rapidly cools below the glass transition temperature (T_g_) after extrusion^3^ leading to poor interlayer adhesion as well as warping.

Interdiffusion between two polymer slabs/layers is a classic problem. In the early 1980’s, it was studied in relation to polymer welding^4–7^. Interest renewed in this area in the mid-2000’s related to self-healing materials^8–12^. As a result, there is a wealth of knowledge that can and has been applied to characterizing and understanding polymer interdiffusion between roads in MatEx^13–18^. Polymer interdiffusion, which derives from random molecular motion of polymer chains, requires that the two surfaces are capable of wetting such that chains are capable of motion across the interface. While there are multiple microscopic models that have been proposed to describe the time dependence of properties during welding/healing^4,5,19,20^, all lead to the conclusion that fracture energy (G_IC_) is proportional to t^1/2^ and fracture stress (σ) and stress intensity factor (K_IC_) are proportional to t^1/4^, where t is the isothermal weld time. This proportionality holds until complete interdiffusion is achieved, at which point bulk mechanical properties are observed.

Despite this robust foundation for understanding what drives weld formation and property evolution in MatEx, we still face hurdles when it comes to accurate prediction of mechanical properties that preclude real-time property prediction. For one, high fidelity modeling is computationally intensive, while lower fidelity models may require simplifications that lead to large inaccuracies. Secondly, the information that we can use as input data is limited to what we can measure, such as temperature (either from thermography^3^ or thermocouples^21–23^) and pressure (e.g., melt pressure^21,22^). Finally, existing modeling methods can predict which conditions will lead to relatively higher or lower strengths/fracture energies, but do not provide information about the minimum conditions necessary to achieve bulk properties. Knowing the requisite process states is particularly important for MatEx, since adding additional energy past the minima necessary to achieve bulk properties can lead to slumping and loss of dimensional accuracy.

This work takes advantage of entanglement theory and molecular dynamics approaches to predict isothermal weld time necessary to achieve bulk properties. A one-dimensional model of heat transfer in a single road width wall is created and validated against both thermography and mechanical testing results for acrylonitrile butadiene styrene (ABS) from Seppala et al.^17^. Various model modifications are investigated to determine which heat transfer considerations are important to predicting properties.

## Theory

### Polymer welding

Interdiffusion between two polymer layers consists of multiple steps. The first step is wetting. Then polymer interdiffusion occurs wherein polymer chains reptate. During this interdiffusion, the mechanical properties of the weld evolve as entanglements form between polymer chains crossing the weld. As stated in the introduction, fracture energy (G_IC_) is proportional to t^1/2^ and fracture stress (σ) and stress intensity factor (K_IC_) are proportional to t^1/4^, where t is the isothermal weld time. Eventually, the interface is microscopically identical to the bulk^4,5,19,20^.

One challenge with applying polymer weld theory to MatEx is that MatEx is a highly non-isothermal process. Time–temperature superposition (TTS) can be used to relate phenomena happening at different time scales and temperatures, or to predict performance of linear viscoelastic materials at temperatures or times that cannot be reached experimentally^24^. For non-isothermal processes such as MatEx, TTS can be a useful principle for normalizing all time–temperature information to equivalent isothermal time at a reference temperature. For amorphous polymers, this relationship can be described using the Williams–Landel–Ferry (WLF) equation:

1 $$\mathrm{log}{a}_{T}=\frac{-{C}_{1}\left(T-{T}_{r}\right)}{{C}_{2}+\left(T-{T}_{r}\right)}$$

where C_1_ and C_2_ are material-specific constants determined by fitting to a master curve, a_T_ is the shift factor, and T_r_ is the reference temperature^25^. This approach has been used to calculate equivalent isothermal weld times for FFF^14–17,26,27^. Additionally, combining TTS with polymer weld theory has shown good agreement between experiments and theory^14,15,17^.

Welding has been investigated via simulations as well as experimentally. Using molecular dynamics (MD) simulations with characteristic time τ, Ge et al. found that the density of entanglements near the interfaces of a weld reached bulk values after 3 × 10^6^ τ of welding^28^. While this was only enough time to create ~ 2 topological constraints per chain with a chain from the opposite side of the interface, this density of topological constraints was sufficient to achieve bulk strength. It is important to note here that topological constraints are interchain contacts in MD between two chains’ primitive paths and, while the spacing between topological constraints is typically 2–3 times smaller than the entanglement length, the densities of entanglements and topological constraints are proportional. Therefore, if we can determine the relationship between 3 × 10^6^ τ and experimental time and temperature, then we will have a prediction of the time necessary to reach bulk entanglement/mechanical properties.

In MD simulations, τ is a characteristic time described by

2 $$\tau =a{\left(\frac{m}{{u}_{0}}\right)}^{1/2}$$

In Eq. (2), a is the molecular diameter and u_0_ is the binding energy. For a polymer chain, a ~ 0.5 nm represents a reasonable interpolymer distance and u_0_ ~ 40 meV gives a binding energy on the order of a glass transition^29^. Using these values gives τ = 0.0342 s at T_g_ for a simple hydrocarbon chain, i.e., polyethylene.

Based on this approach, the time necessary to achieve bulk strength is 3 × 10^6^ τ = 2.83 × 10^5^ s at T_g_. The relationship between tensile strength and weld time can then be described based on the following system of equations:

3 $$\mathrm{t}<2.83\times {10}^{5}\mathrm{ s \; at \; }{\mathrm{T}}_{\mathrm{g}}:\sigma \left(t\right)=\frac{{\sigma }_{bulk}}{{(3M\tau )}^{1/4}}{t}^{1/4}+c$$

4 $$\mathrm{t}\ge 2.83\times {10}^{5}\mathrm{ s \; at \; }{\mathrm{T}}_{\mathrm{g}}:\sigma ={\sigma }_{bulk}$$

σ_bulk_ is bulk tensile strength and c represents the strength associated with wetting. Similarly, the relationship between tear energy (T_c_) and weld time can be described based on the following system of equations.

5 $$\mathrm{t}<2.83\times {10}^{5}\mathrm{ s \; at \; }{\mathrm{T}}_{\mathrm{g}}:{T}_{c}\left(t\right)=\frac{{T }_{bulk}}{{(3M\tau )}^{1/4}}{t}^{1/2}+c$$

6 $$\mathrm{t}\ge 2.83\times {10}^{5}\mathrm{ s \; at \; }{\mathrm{T}}_{\mathrm{g}}{:T}_{c}={T}_{bulk}$$

T_bulk_ is bulk tear energy. Note that the time can be shifted using the WLF equation (Eq. 1) to a different temperature and an integral taken across time and temperature to predict final properties.

### Numerical model

To assess the accuracy of this approach, a simple 1-D thermal model of MatEx was generated. The geometry represents the single road-width wall described in Seppala et al. and Davis et al.^17,26^. Both of these reports used ABS filament as the feedstock. Model inputs (Table 1) were selected to be consistent with the material and printer conditions as described in the experimental work. Specifically, the baseline model parameters were selected to replicate the experimental work wherein Table 1 provides the print geometry, process settings, material properties, and numerical parameters implemented in the simulation. The experimental design included 18 conditions to investigate the role of extruder temperature and print speed while maintaining the print geometry and other process parameters. Simulations were performed to replicate the 18 experimental run conditions and assess the impact of various modeling assumptions with several model permutations as subsequently described.

**Table 1:** Summary of model inputs and printing conditions.

Type of model input	Input	Value
Print geometry	w, road width	0.4 mm
	h, road height	0.3 mm
	r_nozzle_, nozzle outer radius	0.358 mm
	l, layers	16
Process settings	T_ext_, extruder temperature	210–270 °C
	v_print_, print speed	3–100 mm/s
	T_bed_, build platform temperature	110 °C
	T_∞_, environmental temperature	22 °C
	T_∞top_, environmental temperature on top surface	45 °C
Material properties	k, thermal conductivity	0.172 W/m K
	ρ, density	1,080 kg/m^3^
	C_p_, heat capacity	1,670 J/kg K
	T_g_, glass transition temperature	105 °C
	C_1_, temperature sensitivity	4.65
	C_2_, temperature offset	200.9 K
	T_r_, reference temperature	503.15 K
Numerical parameters	h_conv_, convection coefficient	8.5 W/m^2^ K
	Δt, time step	0.001 s
	t_layer_, layer time (speed dependent)	4.33–69 s
	Layer where tear energy was experimentally measured	Between layers 8 and 9

In addition to the simulations performed to replicate the 18 experimental run conditions, other simulations were run in which common print geometry and process setting values were varied. The goal of these simulations was to assess process sensitivity to these process/geometry inputs. Varied parameters and their values are summarized in Table 2.

**Table 2:** Varied parameters and their values.

Input	Values
h	0.1–0.4 mm
T_ext_	210–270 °C
v_print_	3–100 mm/s

A 1-D thermal model of a single road width wall was prepared and run in Matlab R2020a. For simplification, the model assumes that the road cross-section has a rectangular shape and that there is perfect contact between all surfaces, so no thermal resistance is accounted for between roads or between the first road and the build platform. Both of these are common simplifying assumptions in the modeling of MatEx^16,30^, though recent work suggests that thermal contact resistance may play a significant role in the temperature evolution at the interface^31^.

An explicit forward distance method was used for this model. Explicit forward distance methods require small time steps to be stable, but the transient heat transfer of MatEx, in particular FFF, occurs on short time scales, so this is not a significant drawback. Conduction is accounted for between roads and between the first road and the build platform. Natural convection is accounted for between the top and sides of the printed geometry and the environment (air at T_∞_). Forced convection is not considered because it is not present in the experimental system. Radiation is neglected because it has been shown previously to not contribute significantly to heat transfer on the desktop scale^16^. Equations (7–14) describe the base heat transfer model implemented in this work. For printing of the first layer:

7 $$T\left(t=0, layer 1\right)={T}_{0}^{1}={T}_{ext}$$

8 $${T}_{n+1}^{1}={T}_{n}^{1}+4G*\left({T}_{bed}-{T}_{n}^{1}\right)+2H*\left({T}_{\infty }-{T}_{n}^{1}\right)+2M*\left({T}_{\infty }-{T}_{n}^{1}\right)$$

G, H, and M are coefficients for conduction, convection from the sides of the layer, and convection from the top of the layer, respectively.

9 $$G=\frac{k*\Delta t}{\rho *{C}_{p}*{h}^{2}}$$

10 $$H=\frac{\Delta t*{h}_{conv}}{\rho *{C}_{p}*w}$$

11 $$M=\frac{\Delta t*{h}_{conv}}{\rho *{C}_{p}*h}$$

For printing of subsequent layers, the initial temperature of the top layer is T_ext_. Then, the top layer’s temperature is described by

12 $${T}_{n+1}^{top}={T}_{n}^{top}+2G*\left({T}_{n}^{top-1}-{T}_{n}^{top}\right)+2H*\left({T}_{\infty }-{T}_{n}^{top}\right)+2M*\left({T}_{\infty }-{T}_{n}^{top}\right)$$

Layer 1’s temperature is described by

13 $${T}_{n+1}^{1}={T}_{n}^{1}+4G*\left({T}_{bed}-{T}_{n}^{1}\right)+2G*\left({T}_{n}^{2}-{T}_{n}^{1}\right)+2H*\left({T}_{\infty }-{T}_{n}^{1}\right)$$

The temperatures of layers between layer 1 and the top layer (1 < l < top) are given by

14 $${T}_{n+1}^{l}={T}_{n}^{l}+G*\left({T}_{n}^{l-1}-{2T}_{n}^{l}+{T}_{n}^{l+1}\right)+2H*\left({T}_{\infty }-{T}_{n}^{1}\right)$$

This method results in a 1-D model describing the temperature in a cross-section of a single road width wall during and after the printer process—we will refer to this model as the “base” model. Several permutations of this model were also investigated: (A) accounting for less-than-complete contact between roads; (B) higher ambient temperature on top surface to account for presence of extruder; (C) accounting for conduction from the nozzle; and the four combinations of these permutations. These models are described in the following paragraphs.

As a simplifying assumption, the base model assumes full contact across the entire road width; however, single roads form a cross-section that resembles an ellipse^17,32,33^ or a rectangle capped by semicircles^34^. As a better approximation, model variation A added a term W_c_, which accounted for the percent of the domain width where contact occurs between roads (~ 75%)^17^. Equations (7) and (8) are still used to describe printing of the first layer. For printing of the second layer, layer 2’s temperature is described by

15 $${T}_{n+1}^{2}={T}_{n}^{2}+2{W}_{c}G*\left({T}_{n}^{1}-{T}_{n}^{2}\right)+2H*\left({T}_{\infty }-{T}_{n}^{2}\right)+2M*\left({T}_{\infty }-{T}_{n}^{2}\right)$$

Layer 1’s temperature is described by

16 $${T}_{n+1}^{1}={T}_{n}^{1}+4G*\left({T}_{bed}-{T}_{n}^{1}\right)+2{W}_{c}G*\left({T}_{n}^{2}-{T}_{n}^{1}\right)+2H*\left({T}_{\infty }-{T}_{n}^{1}\right)$$

For printing of subsequent layers, the top layers temperature is described by

17 $${T}_{n+1}^{top}={T}_{n}^{top}+2{W}_{c}G*\left({T}_{n}^{top-1}-{T}_{n}^{top}\right)+2H*\left({T}_{\infty }-{T}_{n}^{top}\right)+2M*\left({T}_{\infty }-{T}_{n}^{top}\right)$$

Layer 1’s temperature is described by Eq. (16), and temperatures of layers between Layer 1 and the top layer are given by

18 $${T}_{n+1}^{l}={T}_{n}^{l}+{W}_{c}G*\left({T}_{n}^{l-1}-{2T}_{n}^{l}+{T}_{n}^{l+1}\right)+2H*\left({T}_{\infty }-{T}_{n}^{1}\right)$$

For model variation B, a higher ambient temperature (T_∞top_) is assumed for the top surface. The goal of this variation is to account for the higher temperature in this region due to the local presence of the extruder. Seppala and Migler observed that indirect heating from the extruder increased the temperature of the top layer by up to 3 °C, but measurements of air temperature were not made^3^. For printing of the first layer, Eq. (8) is replaced with

19 $${T}_{n+1}^{1}={T}_{n}^{1}+4G*\left({T}_{bed}-{T}_{n}^{1}\right)+2H*\left({T}_{\infty }-{T}_{n}^{1}\right)+2M*\left({T}_{\infty top}-{T}_{n}^{1}\right)$$

For printing of subsequent layers, the top layer’s temperature is described by

20 $${T}_{n+1}^{top}={T}_{n}^{top}+2G*\left({T}_{n}^{top-1}-{T}_{n}^{top}\right)+2H*\left({T}_{\infty }-{T}_{n}^{top}\right)+2M*\left({T}_{\infty top}-{T}_{n}^{top}\right)$$

Equations (13) and (14) are still used to model the temperatures of layer 1 and intermediate layers, respectively.

Model variation C accounts for conduction from the nozzle to the top surface of the structure as it is being printed. The heat transfer from the nozzle has previously been shown in FEA models to be an important contribution^16^. The general approach here is to create separate expressions for the time when the nozzle is in contact with part of the print and when the nozzle is not in contact with that part of the print. The time that the nozzle would be in contact with a given portion of the print is described by t_c_ as follows:

21 $${t}_{c}=\frac{{r}_{nozzle}}{{v}_{print}}$$

Nozzle radius is used instead of diameter because there is negligible contact in the direction of the print between the printed structure and nozzle in front of the nozzle exit, as shown in Figure SI1. For printing of Layer 1, the initial temperature is still described by Eq. (7). For times between t = 0 and t_c_, Layer 1’s temperature is described by

22 $${T}_{n+1}^{1}={T}_{n}^{1}+4G*\left({T}_{bed}-{T}_{n}^{1}\right)+2H*\left({T}_{\infty }-{T}_{n}^{1}\right)+4G*\left({T}_{ext}-{T}_{n}^{1}\right)$$

For times between t_c_ and t_layer_, Layer 1’s temperature is described by

23 $${T}_{n+1}^{1}={T}_{n}^{1}+4G*\left({T}_{bed}-{T}_{n}^{1}\right)+2H*\left({T}_{\infty }-{T}_{n}^{1}\right)+2M*\left({T}_{\infty }-{T}_{n}^{1}\right)$$

For printing of subsequent layers, the initial temperature of the top layer is T_ext_. Then, the top layer’s temperature is described by the following system of equations:

24 $$\mathrm{For }\left(l-1\right)*{t}_{layer}<t<\left(l-1\right)*{t}_{layer}+{t}_{c}:{T}_{n+1}^{top}={T}_{n}^{top}+2G*\left({T}_{n}^{top-1}-{T}_{n}^{top}\right)+2H*\left({T}_{\infty }-{T}_{n}^{top}\right)+4G*\left({T}_{ext}-{T}_{n}^{top}\right)$$

25 $$\mathrm{For }\left(l-1\right)*{t}_{layer}+{t}_{c}<t<l*{t}_{layer}:{T}_{n+1}^{top}={T}_{n}^{top}+2G*\left({T}_{n}^{top-1}-{T}_{n}^{top}\right)+2H*\left({T}_{\infty }-{T}_{n}^{top}\right)+2M*\left({T}_{\infty }-{T}_{n}^{top}\right)$$

Layer 1’s temperature is described by Eq. (13) and the temperature of intermediate temperatures are described by Eq. (14).

Temperature information from these models is then converted to strength predictions. First, the temperature information up to a given time is converted to an isothermal weld time according to WLF through explicit integration of Eq. (26).

26 $${t}_{weld}\left(t\right)={\int }_{0}^{t}\frac{1}{{a}_{T}}dt$$

Predicted strength is determined by

27 $${\sigma }_{f}\left(t\right)=\frac{{\sigma }_{bulk}}{3\times {10}^{6}\tau }{{t}_{weld}\left(t\right)}^{0.25} \mathrm{for }t<{t}_{bulk}$$

28 $${\sigma }_{f}\left(t\right)={\sigma }_{bulk}\mathrm{ for }t\ge {t}_{bulk}$$

where t_bulk_ is the amount of isothermal weld time necessary to achieve bulk strength, i.e. 2.83 × 10^5^ s at T_g_. Similarly, predicted tear energy is determined by

29 $${T}_{c}\left(t\right)=\frac{{T}_{bulk}}{3\times {10}^{6}\tau }{{t}_{weld}\left(t\right)}^{0.5} \mathrm{ for }t<{t}_{bulk}$$

30 $${T}_{c}\left(t\right)={T}_{bulk}\mathrm{ for }t\ge {t}_{bulk}$$

This approach assumes the theory of time–temperature superposition and adequacy of the WLF constitutive model.

## Results and discussion

### Baseline model

**Figure 1:**
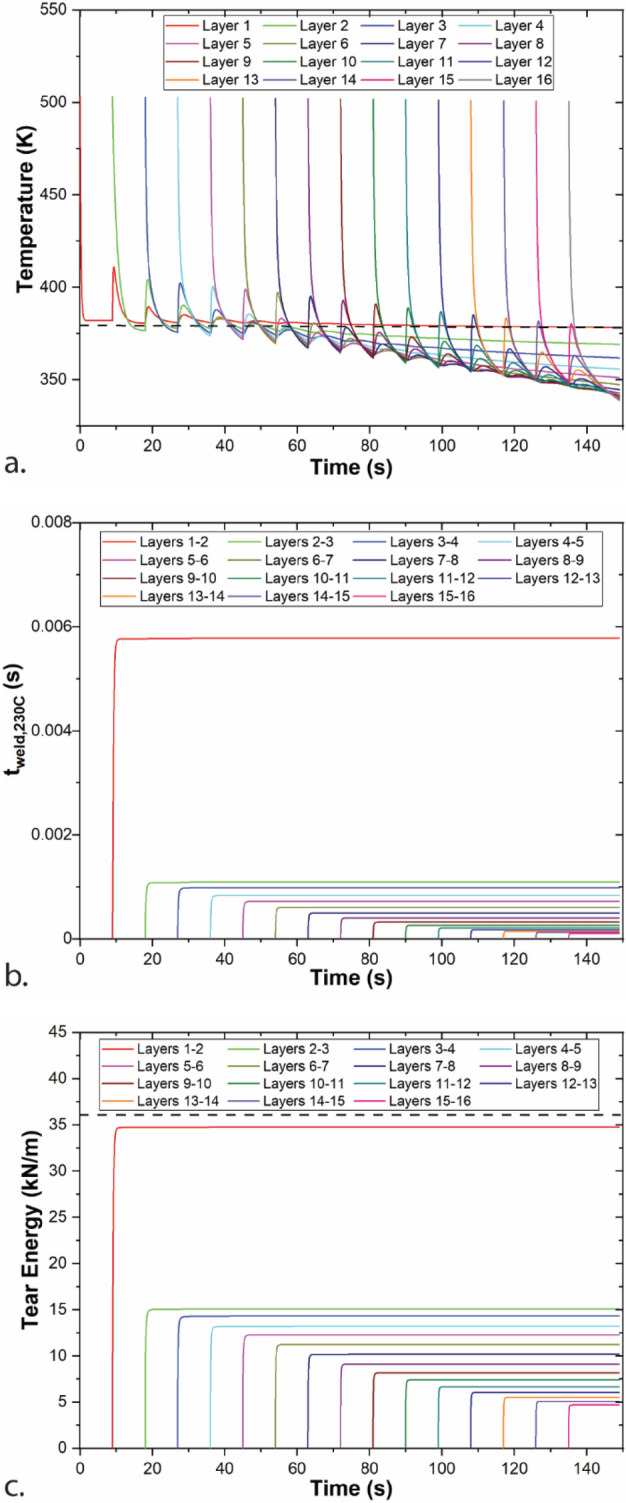
Baseline model results for printing of a single road width wall of ABS for T_ext_ = 230 °C, v_print_ = 30 mm/s, h = 0.3 mm. Geometry and print conditions are based on Ref.^17^. (**a**) Layer temperature; (**b**) weld time at a reference temperature of 230 °C; (**c**) tear energy at interfaces between layers.

Results for the baseline 1D model of printing a single road width wall are shown in Fig. 1. As each layer is deposited, the bulk temperature of that layer appears in Fig. 1a. This temperature rapidly drops, with an average cooling rate of 113 ± 46 °C/s from 230 °C (the extruder temperature) to 130 °C. As the extrudate temperature approaches the temperature of the print surface (print bed for layer 1, previous layer for other layers), the cooling slows. Similar to what has been reported experimentally and with other models, when a new layer is printed, the layer below increases sharply in temperature, and then rapidly re-cools. While layer 1 remains above T_g_ for the entire print, most layers experience little time over T_g_, with time over T_g_ decreasing with increasing layer number (Table 3). Layers closer to the print bed experience multiple segments of welding time where their temperature is above T_g_, with layer 2 going above T_g_ 4 times after deposition, corresponding with the deposition of layers 3–6. Tear energy was experimentally measured in Seppala et al. between layers 8 and 9^17^, which stayed above T_g_ for 6.61 s and 5.61 s, respectively, in the 1D model. Figure SI2 shows temperature, isothermal weld time, and tear energy for the weld.

**Table 3:** Time above T_g_ for each layer and total isothermal weld time of the baseline model (T_ext_ = 230 °C, v_print_ = 30 mm/s, h = 0.3 mm).

Layer	Time above T_g_ (s)	t_weld,230C_ (s)
1	150 (entire model)	
2	5.83 + 7.67 + 8.08 + 7.56 + 5.92 = 35.07	5.78E−3
3	6.05 + 6.79 + 6.35 + 4.62 = 23.81	1.09E−3
4	6.24 + 6.11 + 4.98 + 0.685 = 18.01	9.83E−4
5	5.72 + 5.19 + 3.57 + 14.48	8.38E−4
6	4.95 + 4.31 + 2.22 = 11.47	7.24E−4
7	4.23 + 3.57 + 0.31 = 8.11	6.07E−4
8	3.64 + 2.97 = 6.61	4.96E−4
9	3.15 + 2.46 = 5.61	4.01E−4
10	2.76 + 2.04 = 4.80	3.22E−4
11	2.44 + 1.68 = 4.11	2.61E−4
12	2.19 + 1.39 = 3.58	2.13E−4
13	1.98 + 1.12 = 3.11	1.75E−4
14	1.82 + 0.89 = 2.71	1.46E−4
15	1.70 + 0.67 = 2.37	1.23E−4
16	1.59	1.05E−4

The time for each segment that a layer is above T_g_ is listed and then added together to give the total weld time. Isothermal weld time between layers n and n − 1 is evaluated at a reference temperature of 230 °C.

The time–temperature information for each layer can then be used to calculate weld times at a reference temperature as shown in Fig. 1b for a reference temperature of 230 °C, which was the extruder temperature. All weld times are well below 0.01 s at 230 °C and no appreciable amount of additional weld time is added after the first 1–2 s a weld exists. Weld times do not increase substantially during the segments of time where a layer hops above T_g_ because the times are short and the temperatures remain relatively close to T_g_. Weld times are calculated based on Eqs. (1) and (26). Since temperatures are proportional to the log of the shift factor and weld time is calculated by integrating across the inverse of shift factors, the contribution of time over T_g_ to weld time decreases exponentially as temperature approaches T_g_. While the heating and cooling experienced by lower layers as additional layers are added does not increase weld times, it may lead to additional residual thermal stresses.

Isothermal weld times were then used to predict tear energies, which are shown in Fig. 1c. Similar to weld time, over 99% of tear energy evolves over the first second of welding. These results highlight the short time scales for mechanical properties to form in material extrusion additive manufacturing. Weld time and tear energy for the interface between layers 1 and 2 are substantially higher than for all other interfaces, which is due to the proximity of the heated printed bed that maintains layer 1 at an increased temperature.

**Figure 2:**
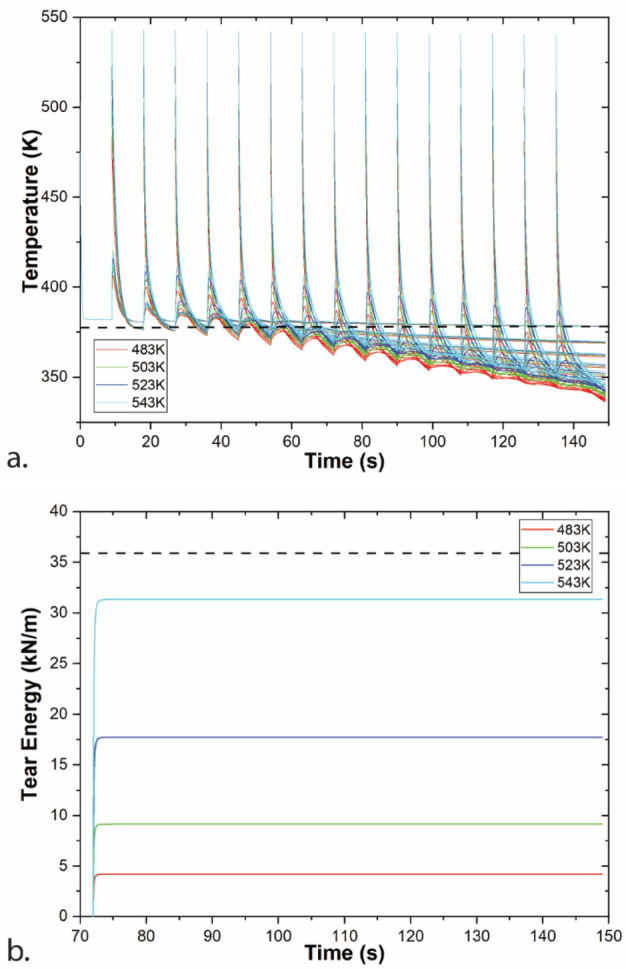
Modeling of a single-road width wall printing at a range of T_ext_ with v_print_ = 30 mm/s, h = 0.3 mm. (**a**) Temperature of all layers at four values of T_ext_; (**b**) tear energies based on the thermal profiles for the weld between layers 8 and 9 for four values of T_ext_.

The effect of extruder temperature is shown in Fig. 2. Increased T_ext_ leads to higher temperatures of the printed structure and higher tear energies. These results are consistent with the literature for ABS, where increased T_ext_ leads to improved welding and higher tear energy^17,26,32^. Extrusion at 270 °C (543 K) leads to a predicted tear energy in the middle of the printed structure (between layers 8 and 9) that reaches 31.3 N/m, 87% of bulk tear energy.

**Figure 3:**
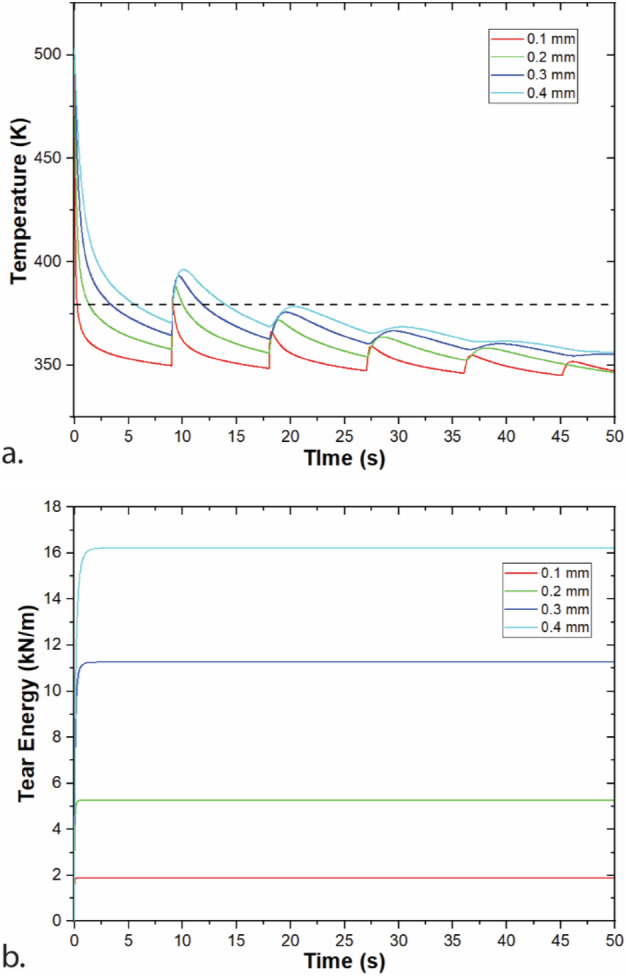
Modeling of a single-road width wall printing at a range of layer thicknesses with T_ext_ = 230 °C, v_print_ = 30 mm/s. (**a**) Temperature of the layer printed at a height of 2.4 mm (layer 24 for 0.1 mm, layer 12 for 0.2 mm, layer 8 for 0.3 mm, and layer 6 for 0.4 mm); (**b**) tear energy for the weld at 2.4 mm. Note that time represents start of layers at same height.

Figure 3 shows the effect of layer thickness on temperature and tear energy at the same height of the printed structure. Temperatures and tear energies are higher for larger layer thickness values. These differences likely result from the longer print time required to reach the same height for smaller layer thicknesses. This additional print time gives the printed structure an opportunity to cool further that is also coupled with a lower characteristic cooling time than thicker layers. Indeed, cooling time of a slab is proportional to the square of the wall thickness. Smaller layer thicknesses enable higher resolution printed structures, so these results highlight the balance between properties needed in material extrusion structures. Interestingly, Coogan and Kazmer reported decreasing bond strength with increasing layer thickness for micro tensile bars laser cut from single road width walls^32^. This trend is attributed to a combination of decreased bond widths and reduced contact pressure^33,35^ for larger layer heights, neither of which this thermal model accounts for.

**Figure 4:**
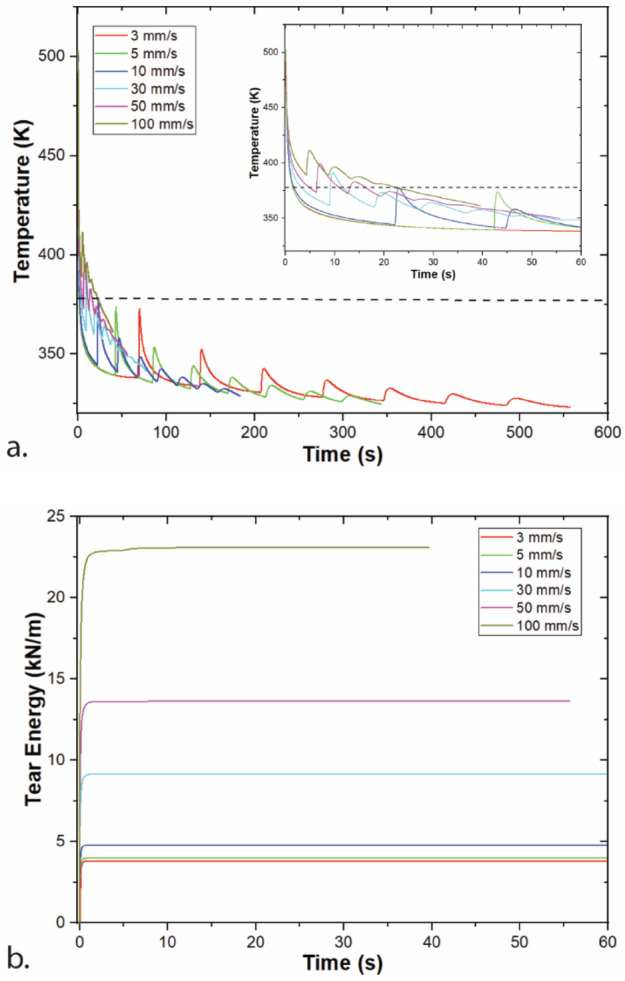
Modeling of a single-road width wall printing at a range of v_print with_ T_ext_ = 230 °C, h = 0.3 mm. (**a**) Temperature of layer 9, with inset showing the first minute after printing; (**b**) tear energy for the weld between layers 8 and 9. Note that time shifted such that t = 0 is the start of layer 9 printing.

**Figure 5:**
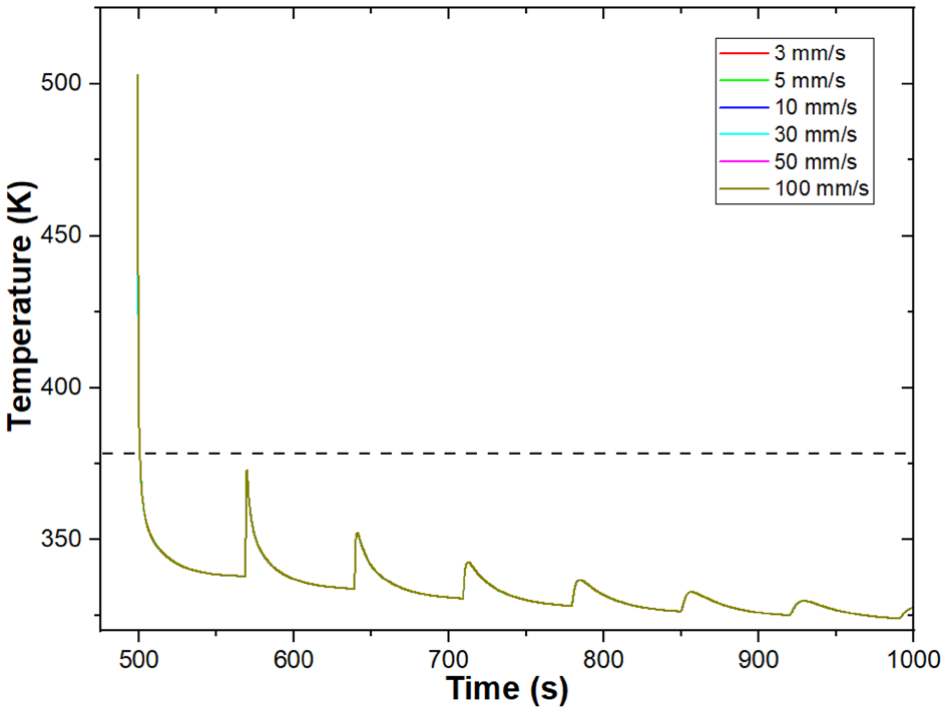
Modeled temperature of layer 9 of a single-road width wall printing at a range of v_print_ and a constant layer time of 70 s. Wall was modeled with T_ext_ = 230 °C and h = 0.3 mm.

Modeling results for a range of print speeds are shown in Fig. 4. Increasing print speed results in shorter layer times, so printing finishes earlier (40 s after layer 9 is printed for v_print_ = 100 mm/s vs. 557 s after layer 9 is printed for v_print_ = 3 mm/s). Since layer times are shorter, the lower layer is warmer when the next layer is printed, leading to higher road temperatures, and, therefore, higher tear energies. When the effect of layer time is controlled for, temperatures are indistinguishable across print speeds, as shown in Fig. 5. These results indicate that the effect of print speed can be well accounted for from layer time. Layer time was first presented as important within large scale material extrusion (big area additive manufacturing, or BAAM)^30,36,37^ and has also been acknowledged as important to the desktop scale material extrusion represented by FFF^2,38,39^. Since increasing layer time leads to lower tear energy in this system, efforts should be made when planning an ABS print to shorten the layer time when practical. However, such process planning will likely lead to printed structures with more, shorter layers. Increased distance from the print bed decreases tear energy as well, so these two concepts should be balanced.

### Model validation

**Figure 6:**
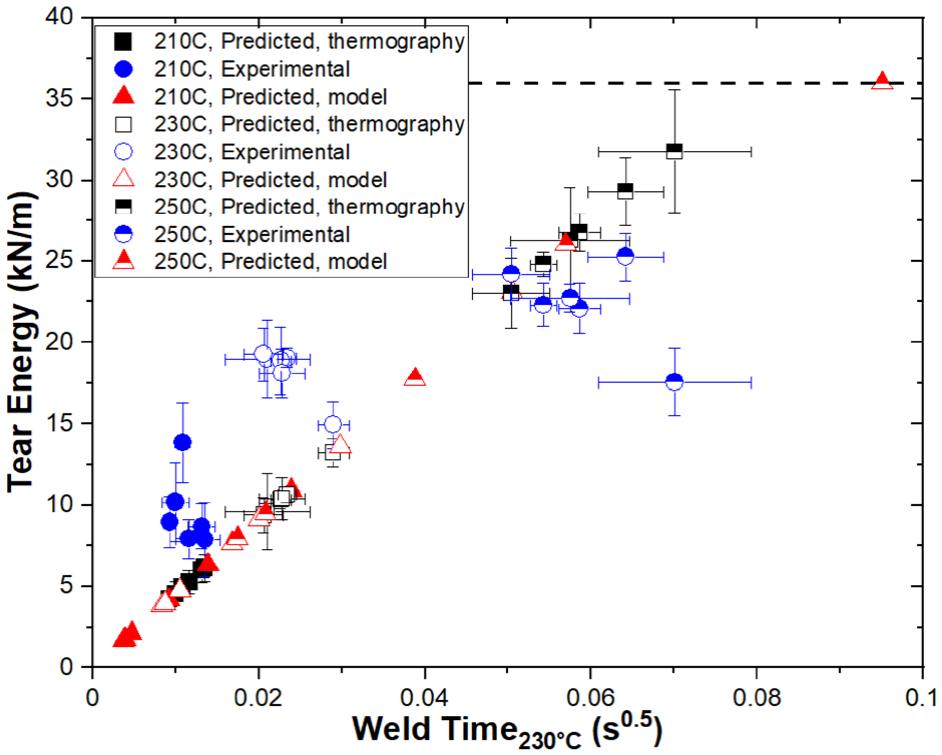
Validation of the model based on tear energy as a function of the square root of isothermal weld time at 230 °C. Tear energies reported include experimental values (blue circles), values predicted from thermography (black squares), and values predicted by the base model (red triangles). Experimental results and thermography data used for prediction are from Ref.^17^. Error bars represent one standard deviation.

To assess the accuracy of the model, results were compared to experimental values of tear energy that were provided in Ref.^17^ as well as tear energies that were calculated based on experimental thermography. In Fig. 6, tear energies are plotted as a function of the square root of isothermal weld time at 230 °C. By definition, tear energy values predicted from thermography and the model scale with the square root of weld time. A similar trend is observed in the experimental tear energies, although some scatter is observed. The deviation from the trend may be due to variations in thermal history or weld width along the weld, or could also arise from compositional variations at the weld^40^ or chain alignment at the weld^41^. Lower extrusion temperatures (T_ext_ = 210 °C and 230 °C) lead to tear energies greater than would be predicted based on weld time, while T_ext_ = 250 °C is better predicted by weld time, and even has one observation that is noticeably underpredicted by weld time. One possible reason for this difference is that thermography is performed on a surface, so if that surface is cooling more rapidly than the interior, thermography may underpredict weld temperatures and tear energies. However, since the thermography does not consistently underpredict tear energy, additional phenomena, such as those previously mentioned, also play an important role in welding. Differences between tear energies predicted from thermography and the model are harder to parse based on Fig. 6, so they are next investigated in greater detail.

**Figure 7:**
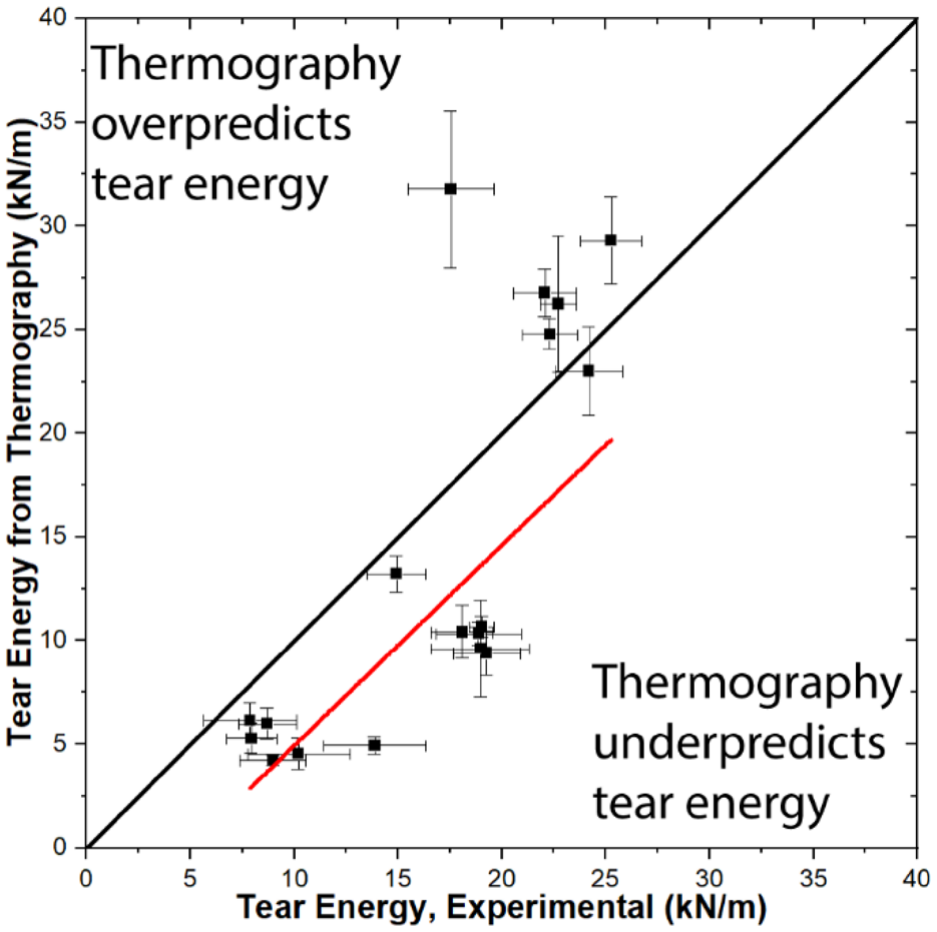
Tear energy from thermography plotted as a function of experimentally measured tear energy. Results are from Ref.^17^. Error bars represent standard deviation.

In Fig. 7, tear energy predicted from thermography and experimental values of tear energy are plotted for each print condition. Comparison of experimental and modeled tear energy values allows for analysis of the approach taken to derive tear energy. In general, a positive correlation is observed between experimental tear energy and thermography-calculated tear energy. R^2^ = 0.663 for a linear fit to the data, so the correlation is present but not especially strong. Values below the x = y line correspond with thermography underpredicting tear energy, while values above the line correspond with thermography overpredicting tear energy. Conditions that lead to lower (< ~ 20 kN/m) tear energies tend to be underpredicted by thermography, while conditions leading to higher (> ~ 20 kN/m) tear energies tend to be overpredicted by thermography. More specifically, thermography underpredicts tear energies for T_ext_ = 210 °C and 230 °C and overpredicts tear energies for T_ext_ = 250 °C. These observations suggests that the deviation is not a function of shear-induced chain alignment. As an alternative, Collinson et al. observed fewer rubber particles in the weld region than in the bulk^40^. These results would be consistent with decreasing migration of rubber particles to the extrudate center with decreasing viscosity (increasing T_ext_). However, the effects of viscosity on particle motion and distribution within viscoelastic confined fluids are complex, especially with soft particles like a rubber, so it’s unclear what real particle distributions would be^42,43^. Davis et al. did not observe differences in road or weld dimensions for ABS extruded at these three T_ext_ values, so dimensional differences likely do not contribute to this difference in prediction ability of thermography^26^.

We hypothesize that the lower-than-predicted tear energies at T_ext_ = 250 °C are due, at least in part, to the fact that thermography on a surfaces does not capture real-time faults and variations in dimensions or temperatures. As we recently reported, real-time differences between input extrudate temperatures and flow rates can be substantial due to physical considerations including limitations in hot end thermal capacity, drool, and the intermeshing of drive gears^22^. Differences in temperature and flow rate will affect the quality and dimensions of a weld. For conditions where thermography overpredicts experimental tear energies, it is likely that minor faults occurred in printing that led to reductions in tear energy.

The two lower populations in Fig. 7 correspond to thermography underpredicting the tear energies at T_ext_ = 210 °C and T_ext_ = 230 °C, which could be due to the surfaces cooling more rapidly than the center in these circumstances. Since the relationship between temperature and isothermal weld time is exponential, even short amounts of time at slightly higher temperatures above T_g_ can appreciably affect t_weld_, and therefore tear energy.

**Figure 8:**
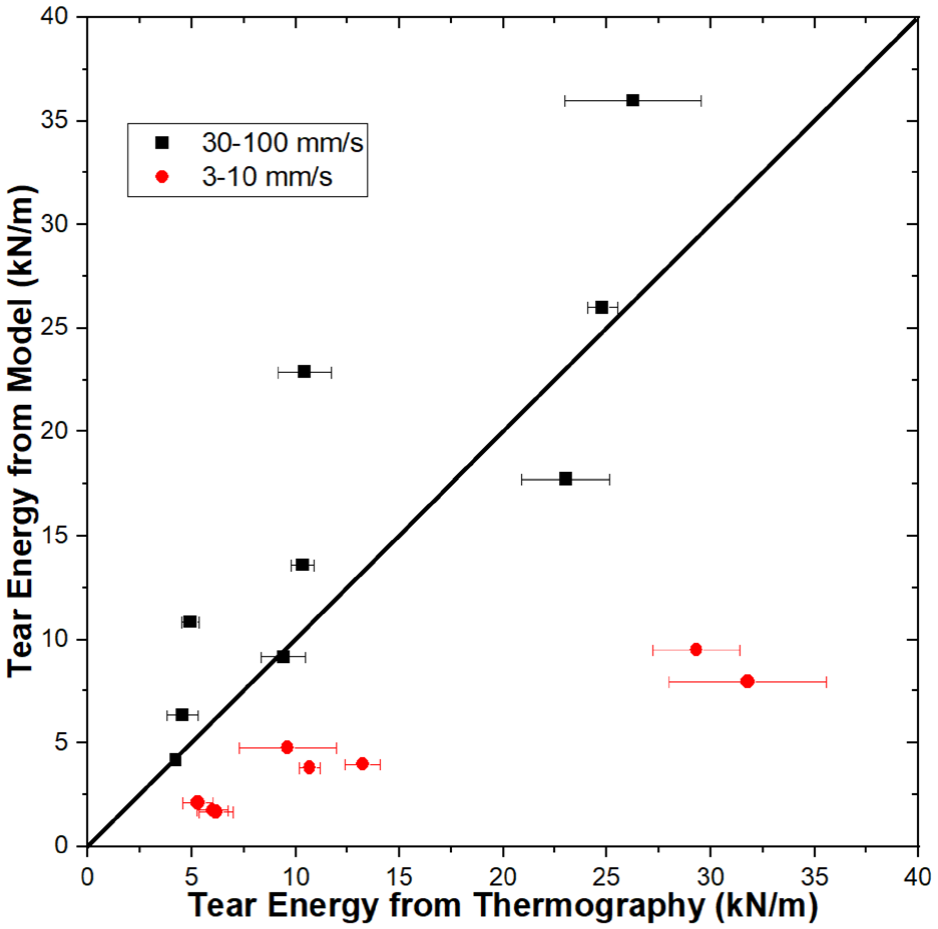
Tear energy from the base model plotted as a function of tear energy calculated from thermography data. Results are from Ref.^17^. Error bars represent standard deviation.

The same approach was taken to determine tear energies from thermography and the thermal model such that comparison of these values provides validation of the thermal model. In Fig. 8, tear energy values from the base model are plotted as a function of tear energy values obtained from thermography. We observe a general positive trend (slope = 0.679) with a low correlation (R^2^ = 0.464). However, print speeds of 30–100 mm/s show a much better fit (slope = 0.977, R^2^ = 0.726) than lower print speeds of 3–10 mm/s (slope = 0.261, R^2^ = 0.905). The agreement between the model and thermography depends on print speed, with the model tending to underpredict tear energy for lower print speeds and showing reasonably good predictive power at higher print speeds.

### Model variations

**Figure 9:**
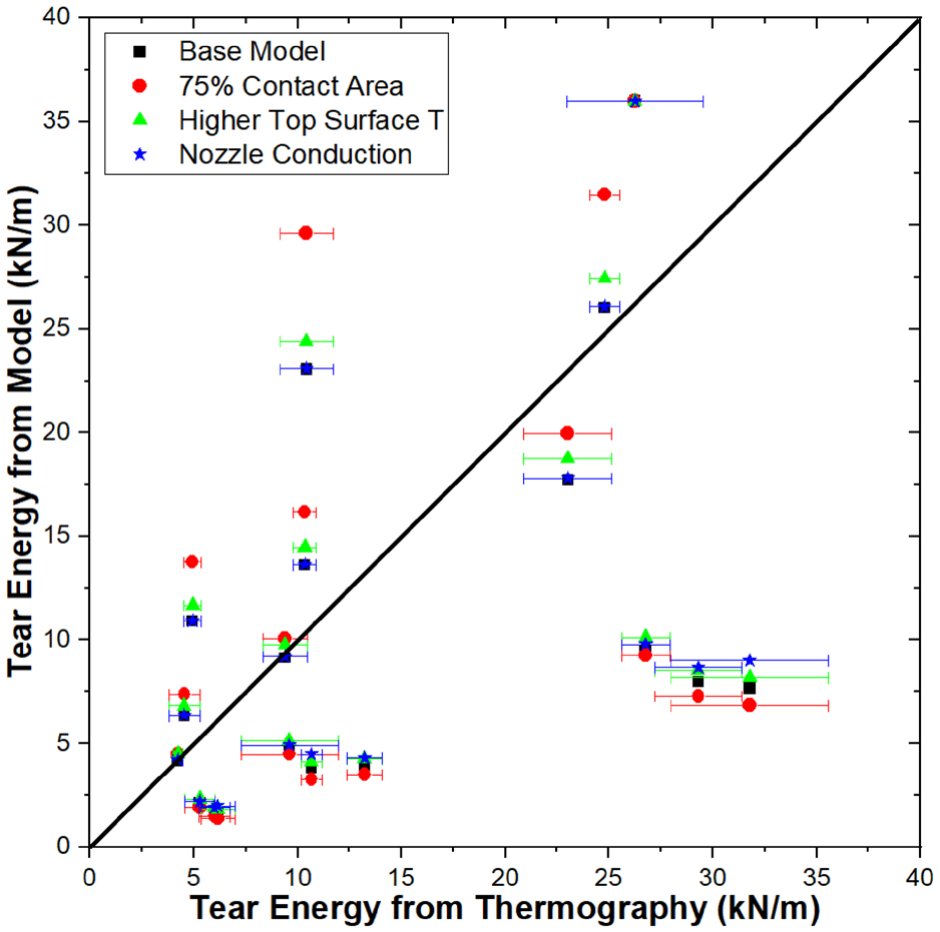
Tear energy from the base model as well as three modifications (75% contact area, higher top surface temperature, and accounting for nozzle conduction) plotted as a function of tear energy calculated from thermography data. Thermography data are from Ref.^17^. Error bars represent standard deviation.

The results from comparing the base model to thermography indicate that the model fails to capture some thermal phenomenona that are more significant at slower print speeds. Three possibilities were considered: A—effect of contact area between layers; B—conduction between the nozzle and print surface; C—convection between the hot end and print surface. Modifications to the base model were implemented to account for each of these possibilities, and results are shown in Fig. 9.

The base model assumes that each layer is a rectangular prism with full contact to the adjacent layers. However, roads of printed structures extruded through nozzles of circular cross-section will exhibit cross-sections that can be described geometrically as ellipses or rectangles capped by semicircles^17,32–34^. Cross-sections from Davis et al. are consistent with this description, with ~ 75% contact between layers^26^. Therefore, the first modification that was considered was to change the model to limit the contact area between layers to 75% of the total surface area. This modification reduced the accuracy of the model results for all but one of the conditions, with increases in modeled tear energy for higher print speed conditions and decreases in tear energy for lower print speed conditions. When specimens have higher print speeds, reducing the contact area appears to prevent thermal conduction to lower layers. When specimens have lower print speeds, reducing the contact area reduces the insulative effect of lower layers, leading to slightly lower tear energies. It should be noted that the overall effect of reducing the modeled contact area from 100 to 75% is modest, with an average change of 1.42 ± 1.87 kN/m (13.2 ± 7.5%).

Next, we consider model variation B, which increases the environmental temperature experienced by the top surface of the printed structure from 22 to 45 °C. The rationale for this model variation is that the presence of the hot end will locally increase the temperature. In all cases, increasing the environmental temperature experienced by the top surface increases the predicted tear energy, since a higher environmental temperature would reduce natural convection and result in the weld staying hotter for longer. Since the environmental temperature difference is relatively modest (33 °C), the average increase in tear energy is also modest at 0.56 ± 0.40 kN/m, or 6.9 ± 2.1%. Because this modification increased all of the modeled tear energies, it improved the accuracy 10/18 conditions (all of which were under-predicted by the base model) and reduced the accuracy of 7/18 conditions. The highest predicted tear energy from the model remained at 36 kN/m, which represents completed welding and a bulk value of tear energy.

In model variation C, conduction between the nozzle and the print surface was accounted for as described in the theory section. Accounting for nozzle conduction leaves the weld hotter for longer, with the effect more pronounced for slower print speeds because the nozzle is in contact with the print surface for longer. For print speeds of 3–10 mm/s, model variation C increased the tear energy by 0.45 ± 0.40 kN/m (10.0% ± 6.4%). For print speeds of 30–100 mm/s, model variation C increased the tear energy by only 0.034 ± 0.028 kN/m (0.28 ± 0.21%). While accounting for nozzle conduction does not result in large increases in tear energy, it increases the under-predicted values more than the over-predicted values, indicating this heat transfer mechanism’s importance in FFF. This finding is consistent with our previous work using a finite element analysis approach to model FFF, wherein we found that accounting for conduction between the nozzle and the print surface enabled the model to accurately predict non-monotonic trends in cooling rate to T_g_ and time to T_g_^16^. Overall, accounting for conduction between the nozzle and the print surface increased the tear energy on average by 0.24 ± 0.35 kN/m (5.1 ± 6.6%). This modification improved the accuracy of all under-predicted conditions (12/18), reduced the accuracy of 5/18 conditions, and did not change the highest predicted tear energy. For the conditions where the accuracy was reduced, the accuracy reduction is vanishingly small (0.18 ± 0.08%).

**Figure 10:**
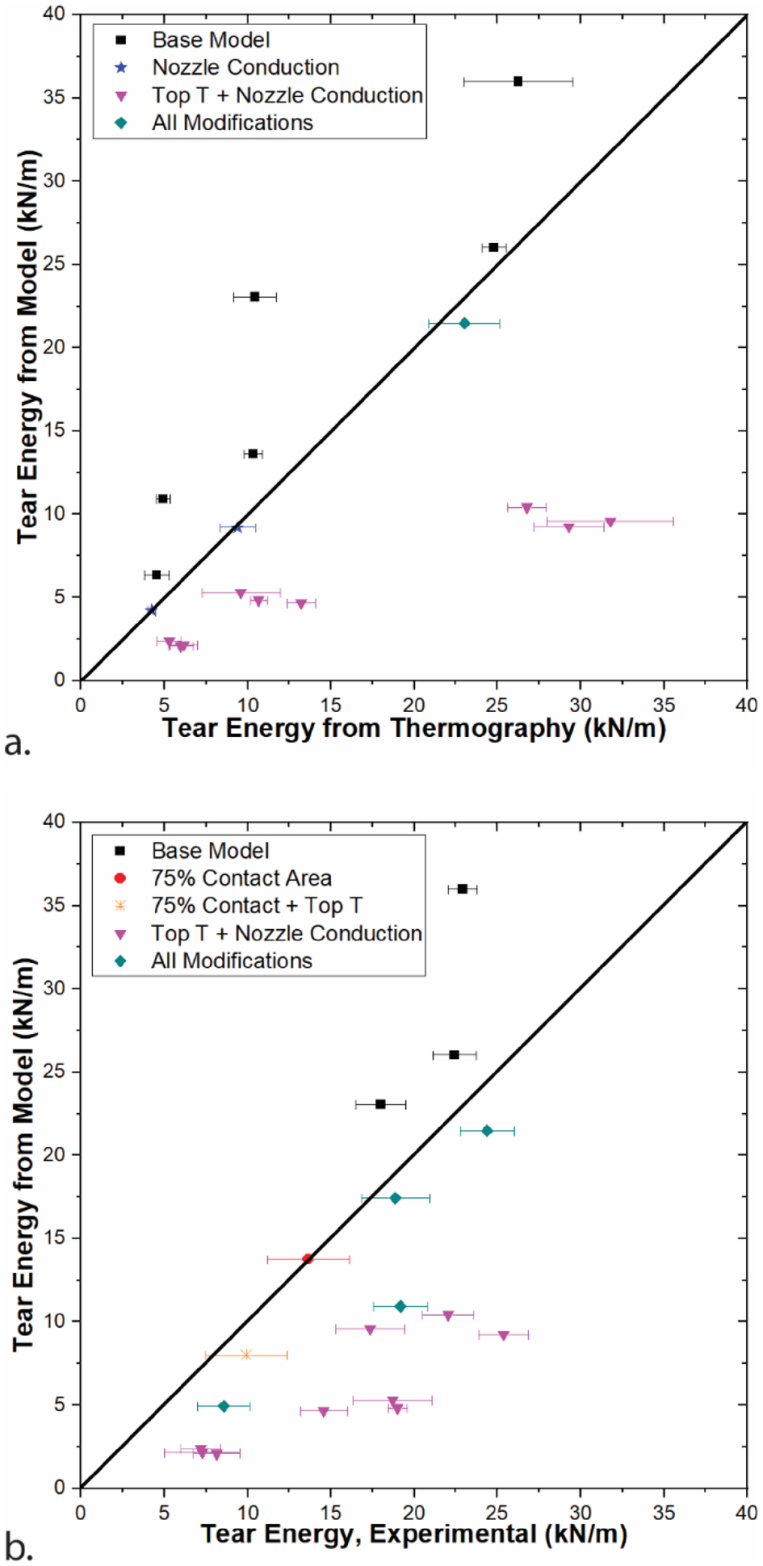
Models that best predict tear energy (**a**) calculated from thermography and (**b**) from experimental measurements of tear energy. Thermography and tear energy data are from Ref.^17^. Error bars represent standard deviation.

Combinations of the three modification were also investigated. For each print condition, the highest accuracy model results are shown in Fig. 10, both when compared to tear energy calculated from thermography and when compared to experimentally-determined tear energy. When compared to thermography, 9/18 conditions show the highest accuracy when a higher temperature of the top surface is combined with nozzle conduction. These conditions correspond to all of the slower print speeds (3–10 mm/s) and are also the observations with the lowest modeled tear energies. For 6/18 conditions, the base model was the most accurate. All of these conditions had print speeds of 50 mm/s or 100 mm/s. All conditions where the base model was the most accurate as compared to thermography were also ones where the model overpredicted tear energy, even if only slightly. As a result, any modification that led to more energy at the weld site decreased the model accuracy. The errors range from 0% (within the standard deviation) to 121%, with the best predicted conditions being at 30 mm/s across extruder temperatures. For 12/18 of the conditions, nozzle conduction is accounted for, highlighting its importance in achieving high fidelity models.

Models were also compared to experimentally-determined tear energies and the model that best predicts experimental tear energy is shown in Fig. 10b. For 13/18 conditions, the best model for thermography was also the best model for experimental tear energy. These conditions include all slower print speeds (3–10 mm/s) for all extruder temperatures as well as 100 mm/s for T_ext_ = 230 °C and all print speeds for T_ext_ = 250 °C. The errors range from 0% (within the standard deviation) to 75%.

## Conclusions

In this work, we used entanglement theory to predict tear energies based on time–temperature information. This approach was able to predict tear energies on reasonable scales with minimal information about the polymer. Such an approach is likely to be applicable to a wide range of amorphous and low crystallinity thermoplastics.

Lower extrusion temperatures, which lead to lower (< ~ 20 kN/m) tear energies, tend to be underpredicted by thermography, while T_ext_ = 250 °C, which leads to higher (> ~ 20 kN/m) tear energies, tends to be overpredicted by thermography. We find that correlations between models and thermography are strong at high print speeds, but low print speeds are poorly predicted by this approach. Three possible modifications, as well as combinations of these modifications, were investigated to improve the quality of models. While the modifications improved model accuracy in some cases, they did not substantially improve the accuracy of the worst performing models and, for overpredicting models, decreased their accuracy.

By comparing thermal models, thermography, and mechanical properties, we find that thermal states are critical but insufficient to predict properties of structures fabricated using desktop scale thermally driven material extrusion additive manufacturing. Models and thermography do not capture real-time faults and variations in dimensions or temperatures that can substantially change mechanical properties in a final structure. This discrepancy motivates ongoing work in developing models and instrumentation for real-time property prediction.

## Supplementary Information

Supplementary Information 1.

Supplementary Information 2.

## Data Availability

Raw data from the model is provided in the Supplementary Information. Thermography is available at https://doi.org/10.1039/C7SM00950J.
