# Supplementary material for: Predicting mechanical properties of material extrusion additive manufacturing-fabricated structures with limited information
Source: Sci Rep. 2022 Aug 30;12:14736. doi: 10.1038/s41598-022-19053-3 (PMC9427823; doi:10.1038/s41598-022-19053-3)
Supplement: Supplementary file 2 — Supplementary Information 2. [file 41598_2022_19053_MOESM2_ESM.docx]

Supporting Information for “Predicting Mechanical Properties of Material Extrusion Additive Manufacturing-Fabricated Structures with Limited Information”


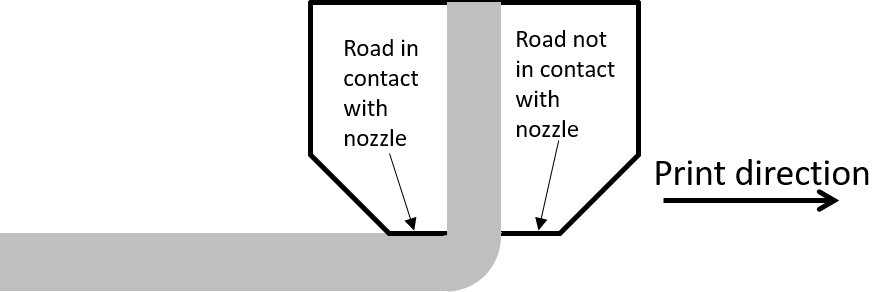


**Figure SI1:** Diagram of printing process showing no contact in the direction of the print between the printed structure and nozzle in front of the nozzle exit.





**Figure SI2:** Layer 9 temperature and isothermal weld time and tear energy for the weld between layers 8 and 9 predicted from the base model.
